# Exploration of fertility and early menopause related information needs and development of online information for young breast cancer survivors

**DOI:** 10.1186/s12905-022-01901-z

**Published:** 2022-08-03

**Authors:** Ellen Marie Sparidaens, Catharina C. M. Beerendonk, Kathrin Fleischer, Willianne L. D. M. Nelen, Didi D. M. Braat, Rosella P. M. G. Hermens

**Affiliations:** 1 Department of Obstetrics and Gynaecology, Radboud University Medical Center, P.O. Box 9101, 6500HB Nijmegen, The Netherlands; 2 Scientific Institute for Quality of Healthcare (IQ Healthcare), Radboud University Medical Center, P.O. Box 9101, 6500HB Nijmegen, The Netherlands

**Keywords:** Information needs, Fertility, Early menopause, Breast cancer, Survivors, Follow up, Women

## Abstract

**Background:**

Approximately half of premenopausal women diagnosed with breast cancer desire to conceive after they finish treatment. Counseling about the risk of infertility prior to cancer treatment has been proven to improve quality of life after cancer treatment. As a result of this, guidelines focus on informing women on this topic prior to treatment. However, it is equally important to provide fertility related information after primary treatment has been completed, when the wish to conceive might become actual. Therefore, the aim of this study was to identify the fertility and early menopause related information needs of young breast cancer survivors and to design, develop and implement online information material with input of stakeholders.

**Methods:**

A phenomenological qualitative study consisting of four phases was performed: identification of information needs through semi-structured interviews from a professional perspective (1) and a patient perspective (2). Exploration of stakeholders perspective regarding development and implementation of online information material (3) and development and implementation of the information material (4).

**Results:**

Professionals indicated that there are no guidelines regarding the provision of fertility related information during cancer survivorship. Survivors reported unmet information needs. Women identified the following as most important information needs (a) fertility preservation options, (b) the risk of menopause or infertility, and (c) long term consequences of early menopause. A wide range of stakeholders involved in breast cancer care were interviewed. Based on their proposed design the information material was implemented on a nationwide website aiming at informing and supporting breast cancer patients.

**Conclusions:**

Fertility and early menopause related information needs of young breast cancer survivors and their professionals were identified. Information material has been designed, developed and nationally implemented. This way, professionals in breast cancer care are provided with an information tool that helps them meet the information needs and preferences of their patients.

**Supplementary Information:**

The online version contains supplementary material available at 10.1186/s12905-022-01901-z.

## Background

Breast cancer is the most common cancer in women aged 15–39 years worldwide [1]. Treatment can be gonadotoxic, leading to temporary or permanent infertility, or premature ovarian insufficiency (POI) later in life [2–4]. Furthermore, young women with a hormone-positive tumor are often treated with adjuvant antihormonal treatment for years after their primary treatment has finished; a period in which they are not able and not recommended to conceive. Whether fertility is regained after the completion of primary and adjuvant treatment depends on factors such as age, type of treatment and cumulative dose of chemotherapy received [2–4].

Due to the social trend of women having children later in life, a growing proportion of women diagnosed with breast cancer does not have completed their family yet [5]. Significant advances in oncology practice over the years have improved breast cancer survival rates. This contributes to the fact that approximately half of premenopausal women diagnosed with breast cancer desire to conceive after they finish treatment [6]. The majority of women experience concerns about the risk of infertility after cancer treatment [7]. These concerns often present as depressive symptoms and a diminished quality of life among young cancer survivors [8–11]. This occurs not only shortly after diagnosis and treatment but has been found to last years beyond the completion of treatment [12]. For women and their partners, the grief associated with the loss of fertility can be as painful an experience as the cancer diagnosis [13].

Younger premenopausal breast cancer survivors report a greater need for information and psychosocial guidance compared to older postmenopausal women regarding topics like fertility and sexuality [14, 15]. Premenopausal women often feel that the information they have received is insufficient or conflicting [16–18]. Professionals do not always feel the necessity of discussing fertility, and women often do not feel encouraged to ask questions about it [16, 19]. This particularly appears to be the case for women who are single at the time of their diagnosis and women who have a poor prognosis [20]. However, receiving information on fertility is important to all premenopausal breast cancer patients [18]. Counseling about the risk of infertility prior to cancer treatment has been proven to significantly improve quality of life after cancer treatment [10, 21].

Current international breast cancer guidelines focus on informing breast cancer patients on the possibility of reduced fertility prior to treatment [22–24]. This is important, because this is the moment a woman needs to be aware of the risks of various treatments for her fertility and the different available fertility preservation options, in order to make well-informed treatment decisions [4, 5, 22–25]. However, concerns about fertility may also arise after cure for cancer in both women who were and were not previously concerned about their fertility [20, 26]. Therefore, it is equally important to provide fertility related information after primary treatment has been completed, when the wish to conceive might change. Since current guidelines lack any recommendations on which information should be provided to breast cancer survivors and how this should be offered [22–24], the present study was designed. Our research question was: what are the fertility and early menopause related information needs of young breast cancer survivors from both professionals’ and patients’ perspectives and how should this information be provided?

## Methods

### Setting

Breast cancer care in the Netherlands is provided in university hospitals, large non-university teaching hospitals and small non-teaching hospitals. Women are primarily diagnosed and treated by a medical oncologist and/or a surgical oncologist. Treatment is multidisciplinary and can also involve a radiation oncologist, radiologist, pathologist, breast cancer nurse, clinical geneticist, plastic surgeon, gynecologist, and a psychologist. The breast cancer nurse coordinates the treatment, is easily accessible for the women, discusses many practical and emotional issues with them and delivers a part of the follow up care. Current Dutch guidelines clarify that all women should be informed about the risk of infertility prior to their treatment and that they should be referred to a specialist in fertility preservation if desired [22], although implementation of this guideline is still inadequate. The specialist in fertility preservation provides information about the risk of infertility or POI and provides counseling about fertility preservation options. A potential delay in breast cancer treatment is considered and discussed with the oncologist. The oncologist is supposed to revisit the subject at the start of follow-up and refer women to a gynecologist again if they have questions [27]. Fertility preservation is centralized in In Vitro Fertilization-centers in the Netherlands.

### Study design

The design of this phenomenological qualitative study, performed in the Netherlands, consisted of four phases: (1) identification of relevant topics concerning fertility and early menopause related information from a professional perspective, (2) identification of fertility and early menopause related information needs from a patient perspective, (3) exploration of needs and preferences of stakeholders involved in breast cancer care regarding development and implementation of online information material, (4) development and implementation of the information. Ethical approval of this study was proposed but was not required according to the local research ethics committee (METC Oost-Nederland file number 2015-1779). All participants signed for informed consent. The COREQ checklist guided the study conduct and reporting.

### Phase 1: identification of relevant topics from a professional perspective

#### Participants

In this phase, the information needs from a professional perspective were identified. Thirteen semi-structured interviews were conducted with a panel of professionals in providing breast cancer care, consisting of five medical oncologists, two surgical oncologists, two gynecologists specialized in fertility preservation, three breast cancer nurses and one specialist in adolescent and young adult cancer care (AYA). Data saturation was reached after the eleventh interview, i.e. no new information items could be added to the topic guide. The experts originated from two university hospitals and three large teaching hospitals throughout the Netherlands via purposeful sampling [28].

#### Data collection and analysis

All interviews were conducted by the first author (EMS) between January and June 2016. The interviews were preferably conducted face to face (n = 11), otherwise by phone (n = 2). The duration of the interviews ranged from 23 to 44 min. Interviews were audio recorded and transcribed verbatim. The topic guide for the interviews was based on a PubMed search on the terms ‘information’, ‘fertility’, ‘menopause’ and ‘breast cancer’. Items regarding fertility and menopause related information for women diagnosed with breast cancer were included in the topic guide. Experts were asked about their experiences concerning fertility and early menopause with women before, during and after breast cancer treatment. For example: which information do they offer women concerning these topics? What questions do women ask? Is there information material available in writing or online? The topic guide for the interviews with professionals is included in Additional file 1: Appendix A. Relevant items concerning fertility or early menopause were identified by EMS, which was supervised by WN. No discrepancies arose during analysis. All items that were identified, were included in the topic guide for the interviews with breast cancer survivors (phase 2).

### Phase 2: identification of information needs from a patient perspective

#### Participants

In this phase the information needs from a patient perspective were identified, using semi-structured interviews. Eligible for inclusion were young female breast cancer survivors, aged 20–45 years old, who had completed their initial treatment, i.e. surgery, chemotherapy and/or radiation therapy, and were currently in their follow up period in one of two participating clinics, i.e. a university hospital and a large teaching hospital. Some women still received antihormonal treatment. Women were consecutively invited to participate during follow up appointments with their oncologist or breast cancer nurse and received written information about the study. Women who agreed to be informed, were contacted by the researcher a few days later, to see if they had further questions and if they wanted to participate. A total of eighteen women participated in an interview, which was determined by data saturation [29]. Data saturation was reached at the sixteenth interview. Two additional interviews yielded no new information. Duration of the interviews ranged from 20 to 60 min. Mean age at breast cancer diagnosis was 35,5 years old, ranging from 21 to 44 years old. Time since diagnosis varied from nine months to twelve years. Most women had been treated with surgery and chemotherapy. Eight out of eighteen participants still had a future childbearing wish at the time of their diagnosis, five of them had chosen fertility preservation. Sociodemographic characteristics are presented in Table 1.

**Table 1:** Sociodemographic characteristics of 18 breast cancer survivors (phase 2)

Characteristic	Number of participants (%)
Time since diagnosis	
< 12 months	1 (6)
12–48 months	6 (33)
> 2 years	11 (61)
Socio-Economic Status (SES)*	
Low	9 (50)
Medium	5 (28)
High	4 (22)
Level of education**	
Low	2 (11)
Medium	6 (33)
High	10 (56)
Treatment received	
Surgery	18 (100)
Radiotherapy	13 (72)
Chemotherapy	17 (94)
Hormone therapy	12 (67)
Currently	8 (44)
Immune therapy	5 (28)
Currently	1 (6)
Relationship status at time of diagnosis	
Married or committed relationship	15 (83)
Single or widowed	3 (17)
Already having children	14 (78)

*According to zipcode area status scores were assigned by the Social Cultural Planbureau of the Netherlands, using the average income, percentage of low income households, percentage of lower vocations and unemployment rates

Low < -1; Medium > -1 and < 1; High > 1

**Low (ISCED 0–2): No education, Basic education, Secondary education

Medium (ISCED 3–4): Lower vocational education

High (ISCED 5–8): Medium vocational education, university

#### Data collection and analysis

The interviews were conducted by the female first author (EMS, MD) between April and December 2017. The interviewer was working as a PhD student and had previously received training in qualitative research techniques and had experience with interviewing techniques. She was not involved in the participants treatment. Interviews were preferably conducted face-to-face (n = 13) in the clinic, but if this was not possible, they were conducted by phone (n = 5). Interviews were audio recorded and transcribed verbatim. Field notes were made by the first author. Each interview started with an explanation of the research goals. The topic guide of the interviews was based on the results of phase 1, i.e., the interviews with the expert panel. It contained open questions, for example on the information women received on fertility and early menopause, current information needs, and online information seeking behavior. Women were encouraged to elaborate on their answers. The topic guide was flexible to allow for new topics that were brought up by participants. The topic guide is included in Additional file 2: Appendix B. At the end of each interview women completed a short sociodemographic questionnaire (Table 1). They were also asked to compose a priority list with a top 5 of most important items concerning fertility and early menopause related information.

Thematic analysis was performed. All transcripts were coded individually by the first author and a research assistant (female medical student) to obtain investigator triangulation [30]. Coded were all items regarding fertility or menopause related information (using Atlas.ti version 8.1.28). Emerging codes were discussed until consensus was reached. If consensus would not be achieved the fourth author (WN) would be consulted, although this was not necessary. Cross-case analysis was conducted, where data from all participants were combined rather than analyzed as individual cases. Individual codes were divided in sub-themes. A constant comparative method was used to interpret the data, continuously reviewing the transcripts [29]. Themes were identified by dominant concepts in the raw data [31].

Analysis of the priority lists included all items that were noted concerning fertility or early menopause. These items received a score based on their ranking on the specific priority list (first ranked 5 points, second one 4 points, third one 3 points, etc.).

### Phase 3: exploration of needs and preferences of stakeholders regarding development and implementation of online information material

#### Participants

The goal of this phase was to identify the needs and preferences of major stakeholders involved in breast cancer care, concerning the design, development and national implementation of information material about fertility and early menopause for young breast cancer survivors. Eighteen stakeholders were interviewed, representing eight patient organizations, seven professional associations in the field of breast cancer care and three medical insurance companies.

#### Data collection and analysis

Stakeholders were asked about their view on nine aspects of design, development and implementation of the information material which were compiled by the research team:

- When should the information be offered?
- Who should offer the information?
- Should every premenopausal breast cancer patient receive the information?
- How detailed should the information be?
- In what format should the information be available?
- Should the information be personalized or interactive?
- What attributes to trustworthiness of information for patients?
- Should the information be integrated in existing platforms?
- How should understandability of the information be ensured?

The interviews were conducted by a research assistant (female medical student). They were audio recorded and transcribed verbatim. The research assistant analyzed the interviews, which was supervised by the first author (EMS). For each of the nine aspects described earlier, different scenarios for implementation were extracted. The scenarios were weighted by both researchers based on six criteria:

- Patient preferences
- Expert preferences
- Financial investment
- Time investment
- Existing literature
- The proportion of patients that is being reached in a scenario

This eventually led to the conclusion of a widely supported recommendation concerning the design, development and national implementation of the information material.

### Phase 4: development and implementation of online information material

In phase 1 and 2 the information needs from both a professional and a patient perspective were identified. Phase 3 led to the conclusion of a widely supported recommendation concerning the design, development and national implementation of the information material. Finally, in phase 4 the information material was developed and implemented based on the results of the previous phases. The information material was developed by the researchers in collaboration with stakeholders interviewed in phase 3. There were several rounds of feedback from both, professionals and patients, before the information material was implemented.

## Results

### Phase 1: identification of relevant topics from a professional perspective

All relevant information items concerning fertility or early menopause that were identified, were included in the topic guide for the interviews with breast cancer survivors, which were performed in phase 2. The topic guide is presented in Additional file 2: Appendix B.

Findings originating from the interviews with professionals were that they stated that the consequences of breast cancer treatment on fertility and possible early menopause were mainly discussed before the start of treatment and only occasionally during and after treatment. Professionals felt like it is their task to initiate the conversation on these topics, since they feel that women struggle to address these topics themselves. Professionals indicated that there are no guidelines on when to discuss these topics, but they do so based on their own intuition. During breast cancer treatment and during the follow up period, both medical and surgical oncologists said that they receive very little questions on fertility and possible early menopause. On the other hand, breast cancer nurses did say that they are asked about these topics by patients, although they feel that they don’t possess the knowledge to properly answer these questions. Both breast cancer nurses and surgical oncologist indicated that they feel the medical oncologist is the designated professional to elaborate on these topics with patients and survivors.

### Phase 2: identification of information needs from a patient perspective

#### Patient interview results

Several information themes were identified from the interview data and are consecutively described below in *italic headings*. Quotes from the interviews are presented in Table 2.

**Table 2:** Interview quotes

Quote number	Participant number	Quote
1	8	‘I brought it up myself with my oncologist, when discussing hormone therapy. I had prepared my questions in advance, because I felt like: This is about my fertility and this is very important to me.’
2	12	‘Very little can be found online. Of course, I know that there are women who had breast cancer and became pregnant after that, often years later. But how that came to be? Did they have IVF or something like that before their treatment? (…) No one tells you how.’
3	14	‘I think it is very important that people can decide for themselves how deep they want to go into the subjects. Because you can fill a website with success stories of people who started a family after breast cancer. But for a lot of people that would be too painful.’
4	10	‘When you are ill, the first priority of your body and your head is: the cancer needs to go. You are not concerned about your fertility, because it does not fit into the picture (..) And I think, during that struggle for survival someone needs to remind you and occasionally tell you ‘this (fertility) matters too’’
5	2	‘What if I quit hormone therapy after three years (instead of five)? What are the consequences? Because it is a preventive treatment. So how much does my risk of breast cancer recurrence increase if I do this? And would it increase my chance of restored fertility?’
6	13	‘What are the consequences? You read about menopausal women that their bones are in worse shape, their hair thins out, skin quality declines. Does that mean that, compared to my mother, when I turn 70 years old I will look a lot older? Or my bones are much more fragile? I do not know and I can not find that anywhere.’
7	11	‘It (menopause) is not something that is visible, and not something you like to share with everyone. Who is proud of going through menopause? (…) So, I think if your relatives or the people close to you know, that would make things easier. Because now they think ‘It is all over, we will celebrate and move on’. But it is not.’
8	7	‘I feel rather lonely. I am around 35 years old. My friends all have children. No one really understands me. (…) I wish I could share my feelings and emotions with someone who is in a similar situation, or to receive information from someone I have not found myself. ‘
9	12	‘That feeling remains. The breast cancer took that (having a second child) away from me. I understand that I had no choice, we needed to start chemotherapy quickly.’
10	17	‘I contacted the hospital myself because it was not endurable. I got mad over nothing. I was cold all the time but suddenly hot. I had no appetite but was gaining serious weight. And I was so unhappy. I had become a completely different person. They really should have informed and guided me on how to deal with that.’

##### Counseling on possible infertility and early menopause

The risk of infertility and early menopause was discussed with most participants at the time of their diagnosis by their medical oncologist, surgical oncologist or breast cancer nurse. Women in this study identified that the most important topics were the risk of diminished fertility, fertility preservation, chemotherapy-induced menopause, contraception, and hereditariness of breast cancer. However, almost half of the women (8 out of 18; 44%) were not satisfied with the amount of information they received and four of them (22.2%) reported not having received any information on the subject.

After completion of their initial treatment, most of the women in this study reported having brought up the topic themselves because they did not feel like they received enough information. They preferred discussing the topic with their breast cancer nurse, medical oncologist or surgical oncologist (Table 2, quote 1). They identified that the most important topics at this point in time were the current fertility status, menopause related symptoms and their treatment, whether to start hormone therapy and contraceptive advice. Only one woman who participated in an interview was referred to a gynecologist.

##### Availability and design of information material

Many participants reported to have consulted other healthcare professionals for support and specialized knowledge, for example their general practitioner, a physical therapist, gynecologist, psychologist or an orthomolecular physician. Furthermore, most women searched for infertility and early menopause-related information online. Search terms that were often used were self-care advices, fertility after breast cancer, menopausal symptoms, side-effects of cancer treatment, sexuality and psychological matters. Women in this study mainly relied on the website of their hospital and the website of the Dutch Breast cancer Association (BVN) to assess reliability of online information. They often described struggling to find reliable information specific to their situation as a young premenopausal woman with breast cancer, or young breast cancer survivor, since most information deemed generic or unreliable to them. (Table 2, quote 2).

Women in this study reported to need written information on fertility and early menopause. They wanted to prepare themselves for hospital visits and read the information again afterwards. This gave them a feeling of being in control and being able to acquire information in their own pace. The preferred medium was a website. The use of a short folder or business card with some information highlights and the link to the website was recommended. Women suggested that a website should use a filter, for example by age or stage of treatment, or use chapters or hyperlinks in the text to avoid women from feeling overwhelmed by the amount of information. They identified that the information should be both concise and detailed, factual and easily accessible. (Table 2, quote 3).

Most participants stated that the information should be developed in collaboration with patient organizations and various hospitals, so that the information is tailored to patient’s needs, perceived as reliable and easy to find. Many participants wanted to be able to ask questions online, preferably to health care professionals. Some women preferred a forum to share experiences with peers. Other women were concerned about a forum, worrying that it would be easily contaminated with overly dramatic stories and unconfirmed claims.

Participants believed that every premenopausal woman who is diagnosed with breast cancer should receive information about fertility and the risk of early menopause, regardless of their age and marital status. Furthermore, they indicated that these topics should be revisited throughout follow up appointments, as they feel their information needs change throughout time. (Table 2, quote 4).

##### Information topics: fertility

Women in this study were in need of personalized information that is specific to their situation as a young woman with a diagnosis of breast cancer. They had questions such as: When should I give up hope of my menstruation cycle returning? When would my body be ready for pregnancy? And when are the risks minimal? Does a pregnancy increase the chance of breast cancer recurrence? Is it safe to stop hormone therapy to try and conceive? (Table 2, quote 5).

Furthermore, women had questions like: Should I be using contraception? If I want to conceive, should we try ourselves? For how long? When can I approach a fertility specialist? When can I use cryopreserved eggs or embryos? Can my child be healthy? Is my chance of a miscarriage increased? Will I be able to breastfeed? What are the alternatives to biological parenthood?

##### Information topics: early menopause

Women in this study indicated that they wanted to understand more about the physiology of menopause. They wanted to learn about hormonal changes and the impact on their body. Most of the participants had experienced or were still experiencing menopause related symptoms. They were in need of information on possible treatment and self-care advices, such as nutrition and exercise. Furthermore, they wanted information on the long term consequences of early menopause. (Table 2, quote 6).

Participants wished for information that also targets their relatives. They felt like their relatives are also in need of support in dealing with the disease. Furthermore, relatives would be able to better support them if they had received more information on their situation. (Table 2, quote 7).

##### Psychosocial impact of unmet fertility and menopause-related information needs

When looking back, some of the women in this study recognized they did not obtain sufficient fertility-related information. For some of them, this would have changed the choices they had made and they were still struggling with that. Not being able to complete their family brought grief. (Table 2, quote 8 and 9).

Furthermore, women reported feeling abandoned after treatment was finished. They needed guidance, also concerning their menopause-related symptoms. (Table 2, quote 10).

#### Priority lists

At the end of each interview participants composed a priority list of the 5 most important information items concerning fertility or early menopause. Some women wrote down less than five items on their priority list, others noted multiple topics per rank. All priority lists were included in the analysis. The sum scores are shown in Table 3. Highest scores were allocated to (a) fertility preservation options, (b) the risk of menopause or infertility, and (c) long term consequences of early menopause.

**Table 3:** Priority lists for information items (n = 18)

Topic	Sum score
Fertility preservation options	54
Risk of menopause/infertility	49
Long term consequences of early menopause	37
Menopausal symptoms	25
Psychological impact of infertility/menopause	10
Self-care advices to improve overall health	9
Referral options	7
Treatment options of menopausal symptoms	7
Options for non-biological parenthood	6
Experiences of peers	5
Dealing with stress	4

All information items received a score based on their ranking on the specific priority list: first ranked 5 points, second one 4 points, third one 3 points, etc.

### Phase 3: exploration of needs and preferences of stakeholders regarding development and implementation of online information material

We identified the needs and preferences of eighteen major stakeholders involved in breast cancer care, concerning information material about fertility and early menopause for young survivors. This led to a widely supported recommendation on nine aspects of design, development and implementation of information material:

#### When should the information be offered?

It was recommended that the information is provided at the moment of diagnosis when also fertility preservation options are being discussed. The information should be revisited when the primary treatment has been completed.

#### Who should offer the information?

The information should be offered to women by the medical oncologist, who has the most expertise on the topic, or by the breast cancer nurse, who is easily accessible to women, has time and attention for psychological issues, and is already frequently involved in follow up care.

#### Should every premenopausal breast cancer patient receive the information?

It was recommended that every women is asked if she is interested in the information material. The actual information material is only provided to women who express their interest.

#### How detailed should the information be?

In general, patients expressed the wish for very detailed information. A proportion of professionals was concerned regarding the feasibility of providing very detailed information, without a professional present to put things into perspective and to provide clarification about what applies to a particular patient. A balance should be found between these visions.

#### In what format should the information be available?

The information should be offered on a website, preferably supplemented with a small paper leaflet to hand over during a consultation, so that a woman can find the information online once she is at home. The information should also be available for women with a language-barrier.

#### Should the information be personalized or interactive?

It was recommended that women have the possibility to personalize the information by using a flowchart. This should help women with lower health literacy to understand the information that is applicable to them. Women who do not prefer personalization should be able to read all the available information. It was not recommended that the information material contains interactive options, since this requires a large investment in both time and finances. Instead, there could be referred to existing online communities.

#### What attributes to trustworthiness of information for patients?

To increase the sense of trustworthiness, the information material should mention the organizations that support the information, for example by placing their logo. Furthermore, these organizations should link to the information material on their website.

#### Should the information be integrated in existing platforms?

To maximize the number of women who are reached with the information material, it was considered helpful if many organizations refer to the information on their website. Hosting the information on an existing platform would be time and cost effective. Moreover, it is an advantage that the information can then be updated by a professional organization, instead of the research group.

#### How should understandability of the information be ensured?

It was recommended that the information material has a simple, professional design. Understandability can be enhanced by providing personal patient experiences, providing video explanations and by designing the information like a funnel, with the options to click for more detail if a woman wishes so.

### Phase 4: development and implementation of online information material

Based on the results of phase 1–3 online information material was developed in collaboration with the nationwide website (http://www.kanker.nl) aimed at informing and supporting cancer patients. This website is supported by all major stakeholders who are involved in Dutch cancer care, namely the KWF Dutch Cancer Society, the Dutch Federation of Cancer Patient Organizations and the Netherlands Cancer Registry. By choosing for this collaboration, it became possible to benefit from the combination of the knowledge concerning information needs gained through this study, and the professional experience in information provision and wide reach of the cancer organization. This way, we were able to implement the information material concerning fertility and early menopause for breast cancer patients on the cancer organization website and ensured easy and wide access and regular updating of the information (information link: Kanker en vruchtbaarheid bij vrouwen).

## Discussion

In our study we investigated the fertility and early menopause related information needs of young breast cancer survivors and their professionals through a phenomenological qualitative study consisting of four study phases. Professionals in providing breast cancer care indicated that there are no guidelines concerning the provision of fertility and early menopause related information, but they address the topic based on their own experience. From a patient perspective it appeared that almost half of the women were not satisfied with the amount of information they received. Furthermore, they struggled to find reliable online information specific to their situation. They needed information on a large variety of topics. Women in this study identified the most important topics as (1) fertility preservation options, (2) the risk of menopause or infertility, and (3) long term consequences of early menopause. Finally, after interviewing a wide range of stakeholders involved in breast cancer care, we executed their proposed design and implementation of information material, being on a nationwide website aiming at informing and supporting cancer patients.

Breast cancer is the most frequently diagnosed cancer among women aged 15–39 years worldwide [1]. Counselling about fertility preservation takes place before the start of oncological treatment [22–24]. This is a phase in which women receive a lot of information in a short time frame. Together with the emotional stress that is caused by the cancer diagnosis, this impairs their ability to remember all information they receive. It is therefore important to provide high quality and easily accessible information to women about the risk of infertility at various points. This includes at the time of diagnosis, throughout the treatment and after treatment has ceased. This way, women can revisit the information at home at the moment fertility related questions arise. Serving these information needs has a positive effect on women’s wellbeing [8–11].

The setting of breast cancer care in the Netherlands differs from most other countries. In the Netherlands, women are primarily diagnosed and treated by a medical oncologist and/or a surgical oncologist, instead of a gynecologist. Women only consult a gynecologist if they are referred for fertility preservation counseling or because of an active wish to conceive. This could possibly impact the experience of unmet fertility and early menopause related information needs among the participants in the current study. However, the current findings largely correspond with previous research. In the current study breast cancer survivors expressed unmet information needs and often reported that they had to bring up the topic themselves during consultations, which is in accordance with previous studies [17, 32]. Literature highlights that young breast cancer patients appreciate information on fertility preservation options, contraception, impact of pregnancy on recurrence of breast cancer, impact of chemotherapy on the health of future children, menopausal symptoms and hormonal therapy [16, 18, 26, 33, 34]. In the current study participants agreed to this and further added information needs on self-care, long term consequences of early menopause, sexuality and information targeted at their relatives. Furthermore, they confirmed the need for information to take home, preferably a pamphlet referring to an online source [18, 35].

The major strength of this study is its multi-phase design. We identified information needs from both, a professional and patient perspective. Furthermore, we consulted various stakeholders in providing breast cancer care concerning the design and implementation of the information material. In this way, we implemented a widely supported information tool that is nationally available for all professionals and patients.

Our study has some limitations. It took a reasonable period to complete all study phases. However, this enabled us to approach the subject from all relevant perspectives and also to implement our recommendations on the nationwide website aimed at informing and supporting cancer patients, which is supported by all major stakeholders involved in Dutch cancer care. Since the start of this study, no comparable initiatives have been carried out, which ensures the relevance of our data to date. Furthermore, it is possible that there has been a selection bias concerning the professionals and patients who chose to participate in the study. This phenomenon is to be expected and underlines the value of providing quantitative data in a larger population through future research.

## Conclusions

Fertility and early menopause related information needs during breast cancer survivorship have been identified and information material has been designed, developed and implemented. The material offers extensive information on these subjects, aimed at young breast cancer survivors. This research has accommodated professionals who provide breast cancer care with an important education tool that helps them provide evidence-based information to the women they are caring for. Future research is needed to evaluate the effect of the new information material and could also explore the added value of such an information tool for other cancer survivors.

## Supplementary Information

**Additional file 1: Appendix A**. Topic list interviews expert panel.

**Additional file 2: Appendix B**. Topic list interviews breast cancer survivors.

## Data Availability

The data analyzed in this study are available from the corresponding author on reasonable request. Contact details: ellenmarie.sparidaens@radboudumc.nl.
