# Supplementary material for: Exploration of fertility and early menopause related information needs and development of online information for young breast cancer survivors
Source: BMC Womens Health. 2022 Aug 3;22:329. doi: 10.1186/s12905-022-01901-z (PMC9351061; doi:10.1186/s12905-022-01901-z)
Supplement: Supplementary file 1 — Additional file 1: Appendix A. Topic list interviews expert panel. [file 12905_2022_1901_MOESM1_ESM.docx]

**Appendix A. Topic list interviews expert panel**

Introduction: explanation of study goals and methods.

Background: in which phase of treatment and/or follow up do you see women with breast cancer?

Do women ask you questions about their fertility or possible early menopause?

If yes:

- When?
- What kind of questions do they ask?
- What do women want to know?
- Do you feel like you are able to answer all questions? What would you need in order to be able to provide better answers?
- Do you address fertility and early menopause related topics?

If no:

- Why do you think these questions are not asked?
- Should these questions be asked?
- Which topics should be addressed? When? Which informations should be provided?
- Do you feel like you are able to answer all questions? What would you need in order to be able to provide better answers?
- Do you address fertility and early menopause related topics?

Do you have access to written information (on paper or digital) about fertility and early menopause aimed at breast cancer survivors, or cancer survivors in general?

If yes:

- Which information?
- Do you refer patients to this information?
- What is the quality of the information?
- Is the information complete? What could or should be added?
- How could this information best be offered to survivors?

If no:

- Is there a need for this information? From yourself of from your patients?
- Which information topics should be included in the information?
- When should this information be offered to survivors?
- How can this information best be offered to survivors?

How do you experience the cooperation between different breast cancer care providers (medical or surgical oncologist, radiation therapist, gynecologist etc)

How do you think we can improve healthcare for breast cancer survivors?
