# Supplementary material for: Exploration of fertility and early menopause related information needs and development of online information for young breast cancer survivors
Source: BMC Womens Health. 2022 Aug 3;22:329. doi: 10.1186/s12905-022-01901-z (PMC9351061; doi:10.1186/s12905-022-01901-z)
Supplement: Supplementary file 2 — Additional file 2: Appendix B. Topic list interviews breast cancer survivors. [file 12905_2022_1901_MOESM2_ESM.docx]

**Appendix B. Topic list interviews breast cancer survivors**

Introduction: explanation of study and interview

Background: which treatments did you have? In which phase of follow up are you currently?

Did you receive information about possible risks for your fertility, before you started treatment?

- If yes: How did you experience this? Which information did you receive? Who provided the information? Was the information sufficient? Were there questions left unanswered? Did you chose for fertility preservation? Why or why not?
- If no: How did you experience this? Did you miss information? If yes: which information? Who should have provided this information to you?

Has your fertility been discussed with you on a later moment?

- If yes: When? How often? What was discussed? By who? Was this sufficient? Why of why not? Were there questions left unanswered? Which ones?
- If no: Did you miss this? What should have been discussed? By who? When?

Have you discussed your fertility or related topics with your general practitioner of other healthcare providers outside the hospital? How did this go?

Do you currently have questions about your fertility or related topics? What questions? Who would you want to discuss this with? What do you want to know? Do you feel like you can address this topics? Why or why not?

Have you ever tried to find information about breast cancer, fertility or related topics? Where did you search this information?

Were there information materials, like websites or pamphlets, that you thought were of good quality?

- If yes: Which? Why do you feel like they are of good quality? Was the information clear? Were all your questions answered?

Would you appreciate receiving information material about fertility and possible early menopause that is aimed at young breast cancer survivors?

- If yes: which information topics should be included? When do you want to receive this information? By who? How would you like to receive it? How should the information look? Should the information be personalized or interactive? Should there be a collaboration with professional organizations or patient organizations? Should there be a collaboration with existing (online) platforms? Which?
- If no: why not?

How do you think we can improve healthcare for breast cancer survivors?
